# Research on the allocation efficiency of integrated medical and older adult care institutions using the Malmquist productivity index

**DOI:** 10.3389/fpubh.2026.1852119

**Published:** 2026-07-01

**Authors:** Lijuan Wang, Xiangchen Kong, Hongxia Zhu, Haozhen Liu, Xihe Yu

**Affiliations:** Department of Social Medicine and Health Management, School of Public Health, Jilin University, Changchun, Jilin, China

**Keywords:** data envelopment analysis, older adult care, Malmquist index, productivity, resource allocation

## Abstract

Rapid population aging has heightened the need for efficient integrated medical and older adult care services in China. Despite policy-driven expansion since 2013, the static efficiency and dynamic productivity performance of integrated medical and older adult care institutions have not been well characterized. This study adopts the Malmquist production index method under the data envelopment analysis (DEA) approach to dynamically assess the total factor productivity (TFP) of 54 integrated medical and older adult care institutions in six provinces (Hebei, Inner Mongolia, Jilin, Shandong, Hubei, and Sichuan) across the eastern, central, and western regions of China from 2020 to 2022 and to analyze the current status and development trends of their productivity. The Malmquist index results showed that total factor productivity increased from 2020 to 2021, with a TFP index of 1.118, but declined from 2021 to 2022, with a TFP index of 0.956, indicating short-term fluctuations in dynamic productivity. These results suggest that the productivity changes of integrated medical and older adult care institutions were not stable across the study period. In terms of regional differences, the comprehensive efficiency and pure technical efficiency in the eastern region are greater than those in the central and western regions. This result may be closely related to differences in economic development, policy support, and the distribution of health care resources. In addition, although some institutions show higher efficiency at the technical level, their comprehensive efficiency fails to reach the optimal level because of irrational scale allocation. Improving the total factor productivity of integrated medical and older adult care institutions is a complex systematic task that not only relies on technological progress but also requires the optimization of resource allocation and scale management.

## Introduction

The World Population Aging 2023 report suggests that the global demographic structure is accelerating and that aging has become a common challenge for many countries (1), with China’s aging process advancing significantly and rapidly. By the end of 2020, China’s older population aged 65 years and above had reached 190.64 million, accounting for 13.5% of the total population and approaching the standard of a deeply aging society (2). This large older population poses many challenges in terms of economic development and social governance. As the size of the older population continues to expand, the traditional model of old-age care has struggled to meet the increasingly diversified and complex demand for old-age care (3). Therefore, to alleviate the old-age care pressure on society and families, the state has actively advocated the development of diversified old-age care models (4) to build a comprehensive and multilevel old-age care service system. Among the many older adult care models, the medical and nursing care model stands out as a key initiative to address the challenges of aging. The medical and nursing care model has become an effective path for alleviating pressure on older adults and for achieving a win–win situation for society, families and older adults because this model can efficiently integrate older adult care and medical resources and provide medical, nursing, health and hospice care while taking care of older adults in their daily lives. Since China first proposed the strategy of promoting the development of combined medical and nursing care in 2013, it has actively explored new modes of cooperation between medical institutions and nursing care institutions, and it has successively issued a series of encouraging and supportive policies to create a favorable policy environment for the development of combined medical and nursing care institutions. The General Office of the Health Commission issued a document to simplify the process of setting up medical institutions within senior care institutions and lowering the threshold of access to encourage more traditional senior care institutions to integrate medical services and expand the scale and coverage of their services. In terms of service quality and supervision, the relevant guidelines emphasize the quality management of health care and nursing care institutions, and they require that medical and nursing care standards be followed and that medical services be integrated into the quality and safety management system. In addition, health care insurance support policies are synchronized to allow qualified in-house medical institutions to be included within the scope of health care insurance payments, reducing the financial burden on older adults and improving the accessibility and affordability of integrated health care services.

In research on older adult care, many scholars have proposed their own views. Maruthappu et al. (5) argued that long-term care service institutions can fully meet the needs of older adults and, on this basis, can constantly improve, optimize and enrich the content of services. At the same time, a closer interactive relationship is formed between service institutions and social institutions, fully mobilizing the strength of social welfare agencies and realizing multiparty cooperation. Glendinning (6) conducted an in-depth study on the effectiveness of care in health care service organizations in the United Kingdom. The findings of the study showed that the fragmentation of the existing older adult care system has been effectively solved by integrating the resources of private doctors, social organizations and public hospitals. Baxter et al. (7) proposed that an integrated care model might increase patient satisfaction and improve perceived quality of care. These international studies suggest that integrated care has become an important strategy for addressing population aging, multi-morbidity, and fragmentation between health and social care systems. However, existing studies have mainly focused on implementation barriers, patient experience, perceived quality of care, and service coordination, while less attention has been paid to the productivity and resource allocation efficiency of institutions providing integrated care for older people. Although DEA and Malmquist index approaches have been applied in health care and long-term care efficiency research (8), evidence remains limited on the dynamic productivity of integrated medical and older adult care institutions, particularly in rapidly aging middle-income settings. Since the implementation of the policy, numerous studies in China have focused on analyzing its operational effects and influencing factors. Du et al. (9) used two-stage data envelopment analysis (DEA) model to evaluate the performance of 30 older adult care institutions in China and proposed that although the efficiency of combined medical and nursing care services has relatively good performance in the two-stage DEA assessment, the allocation of resources still needs to be optimized, and ineffective institutions need to invest in multidimensional inputs, such as operations and management, and so on. At the same time, based on an analysis of the problems existing in the current combination model of medical care and older adult care in China, some researchers (10) have proposed a path for realizing the intelligent combination of medical care and older adult care. Overall, research on service production efficiency can help identify inefficiencies and help managers and policy-makers allocate resources more effectively. Furthermore, an assessment of service productivity can provide a basis for comparisons between different integrated health care organizations (11). Such comparisons can stimulate healthy competition among organizations and continuously improve service levels. Meanwhile, from the perspective of policy formulation, an accurate assessment of service production efficiency helps the government formulate more targeted and effective policies. If a large number of organizations are found to be inefficient in certain aspects due to a shortage of professionals, the government can introduce policies to strengthen the cultivation and introduction of talent in the field of health care integration services.

This study adopts a systematic stratified typical sampling method to determine the research subjects, aiming to analyze integrated medical and older adult care institutions in China comprehensively and in depth. The choice of two provinces in each of the eastern, central and western regions, i.e., Hebei, Inner Mongolia, Jilin, Shandong, Hubei and Sichuan, is based on the reality that there are regional differences in China’s economic and social development levels (12). China is a vast country, and different regions present significant differences in terms of economic development, demographics, policy implementation, and pension needs (13–15). The eastern region is more economically developed (16, 17) and is often innovative and forward-looking in its investment and development model of integrated services, whereas the central region is in a transitional stage, with certain economic strengths and development characteristics. A study of a city in western China (18) revealed that the western region is lagging behind in its economic development level and faces greater challenges in its older adult care services. By selecting representative provinces in these three regions, it is possible to cover the diverse situations of integrated medical and older adult care institutions at different levels of economic and social development, making the research sample more comprehensive and representative (19). This sampling method makes it possible to generalize the results of the study to the whole country. On the one hand, the selection of samples from different regions can reflect the commonalities and characteristics of integrated medical and older adult care institutions under different levels of economic and social development, which helps to uncover universal laws and special strategies for different regions. On the other hand, the random sampling of multiple major types of health care institutions ensures that the sample is diverse in terms of types of institutions and avoids the research limitations caused by a single type of institution. This study aims to provide a comprehensive assessment of the static efficiency and dynamic productivity of China’s integrated medical and older adult care institutions. Specifically, we use DEA to evaluate annual comprehensive efficiency, pure technical efficiency, and scale efficiency, and apply the DEA-Malmquist productivity index to analyze intertemporal changes in total factor productivity (TFP) and its decomposition.

By analyzing the input and output efficiency of integrated medical and older adult care institutions, we determine the differences in the level of productivity and the direction of change in these differences. We more accurately reflect the overall situation of integrated medical and older adult care institutions in China and provide a broadly applicable and practically guiding reference basis for nationwide policy formulation, institutional operation and service optimization. We also offer a comprehensive understanding of the current production efficiency of integrated medical and older adult care institutions to provide scientific, comprehensive and practical guidance for the balanced development of integrated medical and older adult care institutions. By providing dynamic institution-level evidence from China, this study extends the international literature on integrated care by shifting attention from service outcomes and implementation processes to institutional productivity and resource allocation efficiency. The findings may also offer comparative implications for other rapidly aging countries seeking to develop integrated health and long-term care systems under conditions of resource constraints and regional disparities.

This study was designed as a descriptive and dynamic efficiency assessment rather than a second-stage explanatory analysis of socioeconomic determinants. Therefore, regional differences were interpreted as contextual patterns rather than as causal effects of socioeconomic factors.

## Data and methods

### Source of data

From April to July 2023, a systematic stratified typical sampling method was adopted to determine the study population. In this sampling approach, China was first stratified into eastern, central, and western regions according to differences in economic and social development. Two provinces or autonomous regions were then selected from each stratum to represent different regional contexts. Within the selected provinces, institutions meeting the definition of integrated medical and older adult care institutions and having complete data from 2020 to 2022 were included in the sampling frame, and eligible institutions were then selected for analysis. Considering the level of economic and social development in China, two provinces were selected in each of the eastern, central and western regions, namely, the six provinces (regions) of Hebei, Inner Mongolia, Jilin, Shandong, Hubei and Sichuan. The sampled institutions were older adult care institutions that had established or incorporated different types of medical service units, including clinics, infirmaries, rehabilitation hospitals, nursing homes, and general hospitals. In this study, the decision-making unit (DMU) was defined as the individual integrated medical and older adult care institution rather than a regional group of institutions. This choice was based on the operational boundary and management structure of integrated medical and older adult care services in China. In the Chinese context, integrated medical and older adult care institutions commonly provide older adult care services while also establishing or incorporating medical service units, such as clinics, infirmaries, rehabilitation units, nursing homes, or hospital-based service units, within the same institutional setting. These medical service units are not treated as separate hospitals, clinics, or nursing homes in the DEA model; rather, they are embedded in or administratively integrated with the older adult care institution and jointly support the provision of integrated care services. Therefore, each sampled institution functions as a relatively independent operational entity with a unified service objective, resource allocation process, and input–output transformation process. From a DEA perspective, such an institution can be regarded as a DMU because it is the organizational unit that allocates personnel and bed resources and transforms these inputs into older adult care, medical care, nursing, and rehabilitation service outputs. By contrast, using regional groups of institutions as DMUs would aggregate organizations with different ownership, management authority, and service arrangements, which would make it difficult to evaluate institution-level resource allocation efficiency. Therefore, the individual integrated medical and older adult care institution was selected as the DMU in this study. A number of these integrated medical and older adult care institutions were randomly selected from the six provinces as the research sample. The study data were obtained from institutional administrative records and annual statistical reports of the sampled integrated medical and older adult care institutions. After data verification, cleaning, and harmonization, a balanced panel dataset of 54 institutions covering 2020–2022 was constructed. The dataset consisted of aggregated institution-level indicators only and did not include any identifiable individual-level information.

### Study period and robustness considerations

The study period of 2020–2022 was selected because this was the most recent period for which complete, continuous, and comparable institution-level data were available for all sampled institutions. A balanced three-year panel also allowed the Malmquist index to capture two adjacent-period productivity changes, namely 2020-2021 and 2021-2022. Given the relatively short observation window and the potential influence of the COVID-19 pandemic, the technical change component in this study should be interpreted as a short-term relative shift in the observed production frontier rather than as long-term technological progress. To improve the robustness of the estimation, only institutions with complete data for all 3 years were included, the same input and output indicators were applied consistently across years, and productivity changes were reported separately for each adjacent period rather than relying on a single overall mean. Therefore, the findings mainly reflect short-term changes in relative productivity and resource allocation efficiency within the observed sample.

### Treatment of sample heterogeneity

Because the sampled institutions were located in provinces with different levels of economic and social development, potential heterogeneity across decision-making units (DMUs) was considered in the study design and interpretation of the DEA results. First, to ensure the homogeneity required for DEA analysis, all sampled DMUs were integrated medical and older adult care institutions with comparable service functions, and the same input and output indicators were used across institutions and years to ensure consistency in measurement. Second, the DEA-Malmquist model was applied using a common production frontier for the full sample, because the purpose of this study was to evaluate the relative productivity and resource allocation efficiency of institutions under the national integrated medical and older adult care service framework. Third, provincial and regional results were reported and interpreted together with differences in economic development, policy support, and health care resource distribution. Therefore, the efficiency scores in this study should be understood as relative efficiency estimates within the observed sample, rather than as measures completely independent of external environmental conditions.

### Selection of indicators

According to the characteristics of the input and output indicators of integrated medical and older adult care institutions, referring to other studies (20) and considering the availability of data, practicing (assistant) physicians, registered nurses, nursing caregivers, other personnel, and nursing beds were selected as input indicators, and the annual number of totally disabled older adult residents, partially disabled older adult residents, and self-care older adult residents were selected as output indicators. All input and output variables were measured as annual aggregated counts at the institution level and contained no identifiable individual-level information.

The three output variables were defined as the annual number of older adults residing in each institution by ability status. Specifically, “the annual number of totally disabled older adult residents” referred to the number of totally disabled older adults residing in the institution during the year; “the annual number of partially disabled older adult residents” referred to the number of partially disabled older adults residing in the institution during the year; and “the annual number of self-care older adult residents” referred to the number of self-care older adults residing in the institution during the year. These variables reflected the number of residents within each ability category in a given year rather than the duration of stay or person-days of care. This approach was adopted because the administrative records available for all sampled institutions consistently reported annual resident counts by ability status, whereas comparable data on duration of stay were not available across institutions and years. The classification of totally disabled, partially disabled, and self-care older adults was based on the Chinese national standard *Specification for ability assessment of older adults* (GB/T 42195-2022) (21).

### Conceptual framework and terminology

In this study, resource allocation efficiency was used as an overarching concept to describe how integrated medical and older adult care institutions transformed resource inputs, such as human resources and beds, into older adult care service outputs. In the empirical analysis, resource allocation efficiency was evaluated from both static and dynamic perspectives. The static DEA analysis measured comprehensive efficiency, also referred to as overall technical efficiency, which reflected the relative efficiency of each institution in a given year under the observed input–output structure. Comprehensive efficiency was further decomposed into pure technical efficiency and scale efficiency. The dynamic DEA-Malmquist analysis measured total factor productivity (TFP) change over time. TFP reflected intertemporal productivity change rather than static efficiency, and it was decomposed into technical efficiency change and technical change. Technical efficiency change was further decomposed into pure technical efficiency change and scale efficiency change. Therefore, comprehensive efficiency and TFP were not interchangeable concepts; rather, they represented the static and dynamic dimensions of resource allocation performance, respectively.

The Malmquist production index is a dynamic analysis method in the DEA model (22, 23). Using panel data, the DEA-Malmquist framework measures changes in total factor productivity across different periods (20, 24, 25). In this framework, total factor productivity change can be decomposed into efficiency change and technical change, i.e., TFP change = efficiency change × technical change.

In this study, an input-oriented DEA-Malmquist model was used. This orientation was selected because integrated medical and older adult care institutions have more direct managerial control over resource inputs, such as personnel allocation and bed utilization, than over short-term service demand. The number of older adult residents in different ability categories is affected by institutional capacity, admission criteria, care needs, and external demand, and therefore cannot be freely expanded in the short term. Accordingly, the input-oriented model was appropriate for assessing whether institutions could provide the observed level of older adult care services with fewer or better-allocated resources.

The productivity change index of the Malmquist model is calculated as follows:


$$\begin{array}{c} M_{0}^{t+1}={\left[\frac{D^{t}(x_{0}^{t+1},y_{0}^{t+1})}{D^{t}(x_{0}^{t},y_{0}^{t})}\times \frac{D^{t+1}(x_{0}^{t+1},y_{0}^{t+1})}{D^{t+1}(x_{0}^{t},y_{0}^{t})}\right]}^{1/2}\\ =\frac{D^{t+1}(x_{0}^{t+1},y_{0}^{t+1})}{D^{t}(x_{0}^{t},y_{0}^{t})}{\left[\frac{D^{t}(x_{0}^{t+1},y_{0}^{t+1})}{D^{t+1}(x_{0}^{t+1},y_{0}^{t+1})}\times \frac{D^{t}(x_{0}^{t},y_{0}^{t})}{D^{t+1}(x_{0}^{t},y_{0}^{t})}\right]}^{1/2} \end{array}$$

where $M_{0}^{t+1}$ represents the total factor productivity change index, $D^{t}(x_{0}^{t},y_{0}^{t})$ represents the output distance function in period *t*, $D^{t+1}(x_{0}^{t+1},y_{0}^{t+1})$ represents the output distance function in period *t* + 1, $D^{t+1}(x_{0}^{t},y_{0}^{t})$ represents the output distance function when the inputs and outputs of *n* decision-making units in period *t* + 1 are used as the reference set for measuring a certain input and output in period *t* ( $x_{0}^{t},y_{0}^{t}$ ), and $D^{t}(x_{0}^{t+1},y_{0}^{t+1})$ represents the output distance function when the inputs and outputs of n decision-making units in period t are used as the reference set for measuring a certain input and output in period *t* + 1 ( $x_{0}^{t+1},y_{0}^{t+1}$ ).

When $M_{0}^{t+1}$ > 1, it represents an increase in productivity, where the increase = ( $M_{0}^{t+1}$ –1) × 100%. When $M_{0}^{t+1}$ < 1, it represents a decrease in productivity, where the decrease = (1 − $M_{0}^{t+1}$ ) × 100%. When $M_{0}^{t+1}$ = 1, it represents no change in productivity.

The Malmquist productivity change index can be decomposed into 2 components: the first component is efficiency change, i.e., the change from period t to period *t* + 1 relative to the efficiency frontier. If the change in efficiency = 1, there is no change in the efficiency of the decision-making unit. If the change in efficiency >1, there is an increase in the efficiency of the decision-making unit. If the change in efficiency <1, there is a decrease in the efficiency of the decision-making unit. The second component is technical change, i.e., the movement of the efficiency frontier for that the focal decision-making unit from period *t* to period *t* + 1. If the technology change is >1, it means that the movement of technology is positive and progressive. If the technology change is <1, it means that the movement of technology is negative and regressive. If the technology change = 1, it means that the technology has not changed.

## Results

### Static efficiency

Before presenting the static DEA results, the variables reported in Table 1 are defined as follows. Comprehensive efficiency refers to the overall technical efficiency of each decision-making unit (DMU) under the DEA model and reflects the combined effect of managerial efficiency and scale efficiency. Pure technical efficiency refers to the efficiency attributable to institutional management and service capability after excluding the influence of scale. Scale efficiency reflects whether the institution is operating at an optimal scale and is calculated as the ratio of comprehensive efficiency to pure technical efficiency. The type of returns to scale in Table 1 was not assigned in advance or applied differently across institutions; rather, it was determined from the DEA results for each institution in each year as a diagnostic indicator of scale status. CRS refers to constant returns to scale and indicates that the institution is operating close to the most productive scale size. IRS refers to increasing returns to scale and suggests that the institution is operating below the optimal scale, so moderate expansion of inputs may lead to more than proportional increases in outputs. DRS refers to decreasing returns to scale and suggests that the institution may be operating above the optimal scale, so further expansion of inputs may lead to less than proportional increases in outputs. Therefore, the different types of returns to scale reported in Table 1 reflect differences in the scale status of institutions, rather than the use of different DEA models for different institutions.

**Table 1:** DEA-based efficiency analysis of 54 integrated medical and older adult care institutions from 2020 to 2022.

DMU	2020				2021				2022			
	Comprehensive efficiency	Pure technical efficiency	Scale efficiency	Type of returns to scale	Comprehensive efficiency	Pure technical efficiency	Scale efficiency	Type of returns to scale	Comprehensive efficiency	Pure technical efficiency	Scale efficiency	Type of returns to scale
1	1	1	1	CRS	1	1	1	CRS	1	1	1	CRS
2	0.737	0.772	0.956	IRS	0.752	0.786	0.956	IRS	0.605	0.664	0.911	IRS
3	1	1	1	CRS	1	1	1	CRS	0.517	0.673	0.769	IRS
4	1	1	1	CRS	1	1	1	CRS	1	1	1	CRS
5	1	1	1	CRS	1	1	1	CRS	1	1	1	CRS
6	0.398	0.971	0.41	IRS	0.669	0.971	0.689	IRS	0.811	0.944	0.858	IRS
7	0.958	0.975	0.982	DRS	0.644	0.665	0.968	DRS	0.255	0.293	0.869	IRS
8	1	1	1	CRS	1	1	1	CRS	1	1	1	CRS
9	1	1	1	CRS	0.975	0.978	0.998	IRS	0.678	0.679	0.998	DRS
10	0.884	1	0.884	DRS	0.863	1	0.863	DRS	0.684	0.771	0.888	DRS
11	1	1	1	CRS	1	1	1	CRS	1	1	1	CRS
12	0.55	0.751	0.733	IRS	0.558	0.599	0.932	IRS	0.687	0.701	0.981	IRS
13	0.813	0.926	0.878	DRS	0.717	0.965	0.743	DRS	0.793	1	0.793	DRS
14	0.544	0.588	0.926	IRS	0.624	0.643	0.971	IRS	0.638	0.665	0.961	IRS
15	0.553	0.895	0.618	IRS	0.566	0.894	0.633	IRS	0.853	0.907	0.94	IRS
16	1	1	1	CRS	0.895	0.901	0.994	IRS	0.711	0.718	0.99	IRS
17	0.653	0.758	0.862	DRS	0.639	0.827	0.773	DRS	0.494	0.505	0.978	DRS
18	0.718	0.724	0.992	DRS	0.532	0.548	0.97	DRS	0.481	0.487	0.986	DRS
19	0.889	1	0.889	IRS	1	1	1	CRS	0.271	0.762	0.355	IRS
20	0.732	0.868	0.844	DRS	0.579	0.661	0.876	DRS	0.659	0.755	0.873	DRS
21	0.091	1	0.091	IRS	0.392	0.806	0.486	IRS	0.338	0.597	0.567	IRS
22	0.281	0.3	0.936	DRS	0.487	0.557	0.875	DRS	0.46	0.5	0.92	DRS
23	0.643	1	0.643	DRS	0.642	1	0.642	DRS	0.542	0.86	0.63	DRS
24	1	1	1	CRS	1	1	1	CRS	1	1	1	CRS
25	0.834	1	0.834	IRS	0.89	1	0.89	IRS	0.834	1	0.834	IRS
26	0.186	0.609	0.305	IRS	0.186	0.609	0.305	IRS	0.172	0.389	0.442	IRS
27	0.475	0.523	0.908	IRS	0.408	0.453	0.901	IRS	0.281	0.375	0.749	IRS
28	1	1	1	CRS	1	1	1	CRS	1	1	1	CRS
29	1	1	1	CRS	0.973	0.977	0.995	IRS	1	1	1	CRS
30	0.64	0.671	0.954	DRS	0.576	0.646	0.893	DRS	0.513	0.602	0.852	DRS
31	0.641	0.675	0.95	DRS	0.658	0.707	0.93	DRS	0.659	0.702	0.939	DRS
32	0.075	0.507	0.149	IRS	0.2	0.507	0.396	IRS	0.317	0.441	0.719	IRS
33	0.967	0.977	0.989	DRS	1	1	1	CRS	0.884	0.898	0.984	IRS
34	0.823	1	0.823	DRS	0.754	1	0.754	DRS	0.711	1	0.711	DRS
35	0.852	1	0.852	DRS	0.906	1	0.906	DRS	0.857	1	0.857	DRS
36	0.227	0.228	0.994	IRS	0.241	0.241	1	DRS	0.189	0.197	0.96	IRS
37	0.414	0.8	0.518	IRS	0.497	0.7	0.71	IRS	0.466	0.536	0.87	IRS
38	0.412	0.956	0.431	IRS	0.79	1	0.79	IRS	0.897	1	0.897	IRS
39	0.417	0.438	0.95	IRS	0.442	0.454	0.973	IRS	0.521	0.526	0.989	IRS
40	1	1	1	CRS	1	1	1	CRS	0.919	0.925	0.994	IRS
41	1	1	1	CRS	1	1	1	CRS	1	1	1	CRS
42	0.925	0.958	0.966	DRS	0.773	0.824	0.939	DRS	0.646	0.67	0.964	DRS
43	0.836	0.976	0.857	IRS	1	1	1	CRS	1	1	1	CRS
44	1	1	1	CRS	0.89	0.916	0.971	DRS	0.893	0.93	0.96	DRS
45	0.966	1	0.966	DRS	0.924	0.979	0.943	DRS	0.805	0.852	0.945	DRS
46	0.653	0.678	0.962	IRS	0.691	0.705	0.979	IRS	0.703	0.704	0.999	IRS
47	0.939	1	0.939	DRS	0.787	1	0.787	DRS	0.741	1	0.741	DRS
48	0.725	0.736	0.985	IRS	0.606	0.619	0.979	IRS	0.613	0.629	0.974	IRS
49	0.745	1	0.745	DRS	0.655	0.891	0.735	DRS	0.544	0.705	0.771	DRS
50	0.997	1	0.997	IRS	0.777	0.868	0.895	IRS	0.791	0.81	0.977	IRS
51	0.496	1	0.496	IRS	0.496	1	0.496	IRS	0.62	1	0.62	IRS
52	0.733	0.844	0.869	DRS	0.707	0.752	0.94	DRS	0.928	1	0.928	DRS
53	0.882	0.992	0.889	IRS	1	1	1	CRS	0.911	1	0.911	IRS
54	0.843	0.863	0.977	IRS	0.931	0.94	0.99	IRS	1	1	1	CRS

The 54 integrated medical and older adult care institutions were coded as follows: DMU1–DMU11 (Hebei, *n* = 11), DMU12–DMU25 (Shandong, *n* = 14), DMU26–DMU28 (Jilin, *n* = 3), DMU29–DMU37 (Hubei, *n* = 9), DMU38–DMU46 (Inner Mongolia, *n* = 9), and DMU47–DMU54 (Sichuan, *n* = 8).

The results in Table 1 indicate that 14 integrated medical and older adult care institutions had strong DEA efficiency in 2020: DMU1, DMU3-5, DMU8-9, DMU11, DMU16, DMU24, DMU28-29, DMU40-41, and DMU44. Fourteen integrated medical and older adult care institutions had strong DEA efficiency in 2021: DMU1, DMU3–5, DMU8, DMU11, DMU19, DMU24, DMU28, DMU33, DMU40-41, DMU43, and DMU53. In 2022, 11 integrated medical and older adult care institutions had strong DEA efficiency: DMU1, DMU4-5, DMU8, DMU11, DMU24, DMU28-29, DMU41, DMU43, and DMU54. Among them, eight integrated medical and older adult care institutions, namely, DMU1, DMU4-5, DMU8, DMU11, DMU24, DMU28, and DMU41, have strong DEA efficiency for three consecutive years. Their comprehensive, pure technical, and scale efficiencies are all 1, and their scale payoffs are unchanged. These findings indicate that the resource inputs and outputs of these integrated medical and older adult care institutions are in a relatively optimal state, that the resource inputs in terms of scale and technology are fully utilized, and that the outputs are maximized.

Among the other inefficient integrated medical and older adult care institutions, 12 institutions were purely technically efficient in 2020, namely, DMU10, DMU19, DMU21, DMU23, DMU25, DMU34-35, DMU45, DMU47, and DMU49–51. In 2021, 8 integrated medical and older adult care institutions were purely technically efficient, namely, DMU10, DMU23, DMU25, DMU34-35, DMU38, DMU47, and DMU51. In 2022, 9 integrated medical and older adult care institutions were purely technically efficient, namely, DMU13, DMU25, DMU34-35, DMU38, DMU47, and DMU51–53. Among them, DMU25, DMU34-35, DMU47, and DMU51 were purely technically efficient for three consecutive years, indicating that these five integrated medical and older adult care institutions were in a relatively optimal state in terms of management level and service capability. However, their comprehensive efficiency remained ineffective mainly because their institutional scale was not reasonably allocated.

From the aggregated data, the efficiency of integrated medical and older adult care institutions varied across the six provinces. Hebei has the highest efficiency, with an average efficiency value of nearly 0.9, indicating that its performance in resource allocation and operations is relatively excellent. In contrast, Jilin has the lowest efficiency, with an average efficiency value of only 0.523, indicating that there may be more room for improvement in the provision and management of medical and nursing care (see Table 2).

**Table 2:** Efficiency analysis of integrated medical and older adult care institutions in different provinces.

Efficiency	Province						Summary
	Inner Mongolia	Jilin	Sichuan	Shandong	Hebei	Hubei	
Comprehensive efficiency	0.819	0.523	0.769	0.657	0.862	0.631	0.730
Pure technical efficiency	0.870	0.662	0.902	0.802	0.913	0.741	0.833
Scale efficiency	0.944	0.734	0.860	0.830	0.939	0.841	0.872

### Dynamic productivity

The Malmquist productivity index measures productivity change from period *t* to period *t* + 1. A value greater than 1 indicates an improvement, whereas a value less than 1 indicates a decline. The Malmquist index (total factor productivity change, denoted as TFP) can be decomposed into efficiency change (EC) and technical change (TC), i.e., TFP = EC × TC. Furthermore, EC can be decomposed into pure technical efficiency change (PEC) and scale efficiency change (SEC), i.e., EC = PEC × SEC. Table 3 reports the adjacent-period Malmquist index values for 2020-2021 and 2021-2022, whereas Table 4 reports the institution-level Malmquist index values and rankings for the 54 DMUs.

**Table 3:** Malmquist productivity index and decomposition of 54 integrated medical and older adult care institutions from 2020 to 2022.

Time interval	Efficiency change	Technical change	Pure technical efficiency change	Scale efficiency change	Total factor productivity change
2020-2021	1.035	1.080	0.994	1.041	1.118
2021-2022	1.081	0.884	1.002	1.079	0.956

**Table 4:** Malmquist productivity index of integrated medical and older adult care institutions from 2020 to 2022.

DMU	Efficiency change	Technical change	Pure technical efficiency change	Scale efficiency change (SEC)	Total factor productivity change (TFP)	Ranking
1	1.000	0.975	1.000	1.000	0.975	29
2	0.876	1.050	0.917	0.955	0.920	35
3	0.751	1.029	0.848	0.886	0.773	53
4	1.000	0.981	1.000	1.000	0.981	28
5	1.000	1.003	1.000	1.000	1.003	23
6	1.507	0.952	1.015	1.485	1.435	6
7	0.668	0.834	0.689	0.970	0.557	54
8	1.000	1.006	1.000	1.000	1.006	22
9	0.896	0.921	0.915	0.979	0.825	47
10	0.916	0.966	0.914	1.002	0.885	43
11	1.000	1.436	1.000	1.000	1.436	5
12	1.150	0.939	0.996	1.155	1.080	15
13	1.024	0.960	1.010	1.014	0.983	26
14	1.083	1.024	1.072	1.010	1.109	12
15	1.221	0.996	1.051	1.162	1.216	8
16	0.848	0.958	0.862	0.984	0.812	50
17	0.942	0.969	0.928	1.015	0.913	40
18	0.829	0.992	0.826	1.004	0.822	49
19	0.712	1.117	0.933	0.763	0.795	51
20	0.965	1.018	0.915	1.055	0.982	27
21	2.011	1.145	0.782	2.571	2.303	1
22	1.326	1.001	1.269	1.045	1.327	7
23	0.986	0.941	0.938	1.051	0.928	34
24	1.000	1.034	1.000	1.000	1.034	18
25	0.971	1.032	1.000	0.971	1.002	24
26	1.107	0.825	0.969	1.142	0.913	40
27	0.788	0.991	0.894	0.881	0.781	52
28	1.000	1.033	1.000	1.000	1.033	19
29	1.000	1.000	1.000	1.000	1.000	25
30	0.965	0.990	1.001	0.964	0.955	31
31	0.988	1.023	1.016	0.972	1.011	20
32	2.109	1.016	0.948	2.225	2.142	2
33	0.960	1.004	0.988	0.972	0.964	30
34	0.907	1.010	1.000	0.907	0.916	37
35	1.059	0.953	1.000	1.059	1.009	21
36	0.945	0.968	0.963	0.981	0.915	38
37	1.137	0.939	0.857	1.327	1.068	16
38	1.550	1.016	1.024	1.514	1.575	3
39	1.133	0.988	1.117	1.014	1.119	11
40	1.000	0.948	1.000	1.000	0.948	32
41	1.000	0.860	1.000	1.000	0.860	46
42	0.944	0.874	0.953	0.991	0.825	47
43	1.000	1.084	1.000	1.000	1.084	14
44	1.000	0.915	1.000	1.000	0.915	38
45	0.981	0.935	1.000	0.981	0.917	36
46	1.221	0.874	1.189	1.027	1.067	17
47	0.896	0.973	1.000	0.896	0.872	44
48	0.981	0.954	0.976	1.005	0.936	33
49	0.912	0.945	0.889	1.026	0.862	45
50	0.902	1.011	0.913	0.988	0.912	42
51	1.288	0.873	1.000	1.288	1.124	10
52	1.124	1.028	1.032	1.089	1.156	9
53	1.000	1.450	1.000	1.000	1.450	4
54	1.060	1.037	1.045	1.014	1.099	13

On the basis of the Malmquist production index model, MATLAB software was used to calculate the dynamic changes in total factor productivity of 54 integrated medical and older adult care institutions in Hebei, Inner Mongolia, Jilin, Shandong, Hubei, and Sichuan from 2020 to 2022. Specifically, only the 2020-2021 period experienced growth in total factor productivity, while the 2021-2022 period experienced negative growth in total factor productivity. Pure technical efficiency change was 0.994 from 2020 to 2021, indicating a slight decline of 0.6%. Technical change and total factor productivity change were 0.884 and 0.956 from 2021 to 2022, indicating declines of 11.6 and 4.4%, respectively.

From 2020 to 2022, 24 of the 54 integrated medical and older adult care institutions had a total factor productivity index greater than 1, accounting for 44.4% of the sample. Among them, 4 out of 11 institutions in Hebei Province, 7 out of 14 institutions in Shandong Province, 1 out of 3 institutions in Jilin Province, 4 out of 9 institutions in Hubei Province, 4 out of 9 institutions in Inner Mongolia, and 4 out of 8 institutions in Sichuan Province had a total factor productivity index greater than 1. Efficiency change, technical change, pure technical efficiency change, and scale efficiency change were all greater than 1 in five institutions, namely DMU14, DMU22, DMU38, DMU52, and DMU54, whereas these four components were all less than 1 in six institutions, namely DMU7, DMU9, DMU16, DMU27, DMU36, and DMU42.

## Discussion

With the aging of the population, integrated medical and older adult care institutions are playing an increasingly important role in the construction of China’s older adult service system (26). This paper examines the dynamic changes in total factor productivity (TFP) of integrated medical and older adult care institutions in six provinces, namely, Shandong, Hebei, Hubei, Jilin, Inner Mongolia, and Sichuan, through stratified sampling from 2020 to 2022, with the aim of revealing the current status and development trend of their productivity and providing a scientific basis for policy formulation.

The results showed that total factor productivity increased from 2020 to 2021, with a TFP index of 1.118, but declined from 2021 to 2022, with a TFP index of 0.956. This indicates that productivity changes were not stable across the study period. From the decomposition results, efficiency change increased in both periods, with index values of 1.035 and 1.081, respectively, whereas technical change increased from 2020 to 2021 but declined from 2021 to 2022. Pure technical efficiency change was 0.994 from 2020 to 2021, indicating a slight decline of 0.6%. Technical change and total factor productivity change were 0.884 and 0.956 from 2021 to 2022, corresponding to declines of 11.6 and 4.4%, respectively.

In the context of this study, technical efficiency change reflects whether institutions moved closer to or farther away from the best-practice frontier under the existing input–output structure. An increase in technical efficiency change indicates that institutions improved their ability to use available resources, such as physicians, nurses, nursing caregivers, other personnel, and beds, to provide services for older adults with different levels of disability. Therefore, changes in technical efficiency are more closely related to internal management, resource coordination, service organization, and scale adjustment. By contrast, technical change reflects the shift of the observed production frontier over time. In this study, it does not simply refer to the adoption of new medical technologies, but rather to broader changes in the service production environment, including care delivery models, medical-nursing integration capacity, information management, rehabilitation and nursing service capacity, and the external policy and public health environment. The results suggest that although institutions continued to improve their relative efficiency from 2021 to 2022, the decline in technical change offset these gains and led to a decrease in total factor productivity. Therefore, the decline in TFP during this period should be interpreted mainly as a short-term relative contraction of the observed production frontier rather than as a deterioration in internal efficiency alone.

In terms of regional differences, comprehensive efficiency and pure technical efficiency are relatively high in the eastern region, whereas they are relatively low in the central and western regions. For example, the combined efficiencies in Shandong and Hebei Provinces are 0.862 and 0.730, respectively, whereas those in Inner Mongolia and Jilin are 0.631 and 0.523, respectively. This difference may be related to the level of economic development, the strength of policy support, and the distribution of health care resources (27). At the level of specific institutions, the study revealed that only eight institutions have maintained strong DEA efficiency for three consecutive years, and the resource inputs and outputs of these institutions are in a relatively optimal state. Although some institutions perform well in terms of pure technical efficiency, they fail to optimize their comprehensive efficiency because of irrational scale allocation. This finding suggests that improving the efficiency of integrated medical and older adult care institutions not only relies on progress at the technical level but also requires optimization in terms of resource allocation and scale management.

The results of this study show that the total factor productivity of integrated medical and older adult care institutions is affected by a variety of factors. First, changes in the policy environment have a significant effect on the productivity of institutions. For example, the global COVID-19 outbreak in 2020 led to the emergency deployment of medical resources, which affected the normal operational efficiency of institutions (28). Second, the decomposition results suggest that improvements in internal resource utilization alone were not sufficient to sustain TFP growth when technical change declined. This phenomenon may be related to institutions’ insufficient attention to technological investment, especially investment in medical technology and information management (29, 30).

In addition, the level of economic development and the demographic structure among regions affect the productivity of medical and nursing care institutions (31, 32). Economically developed regions are usually able to attract more resource inputs, thus improving the operational efficiency of institutions. In addition, densely populated regions have a greater demand for senior care services, which also prompts institutions to optimize resource allocation and service quality.

Although this study was conducted in China, the findings may offer broader lessons for other countries facing rapid population aging and increasing demand for integrated health and long-term care services. The results suggest that improving institutional productivity requires not only expanding resource inputs, but also optimizing the allocation of medical staff, nursing caregivers, and beds. The decomposition of total factor productivity further indicates that internal efficiency improvement and shifts in the production frontier may be driven by different factors, suggesting that policymakers should distinguish institutional management improvements from broader changes in care delivery models, workforce organization, digital management capacity, and external operating environments. In addition, the observed regional differences highlight the need to consider local economic conditions, demographic structure, and health care resource distribution when designing integrated care systems.

### Limitations

This study has several limitations related to the DEA-Malmquist method. First, DEA is a deterministic frontier method, and deviations from the efficiency frontier are treated as inefficiency. Therefore, the estimated efficiency scores may be sensitive to outliers, measurement errors, and random fluctuations in the input and output data. Although the data were verified, cleaned, and harmonized before analysis, potential reporting errors in institutional administrative records could not be completely excluded. Second, DEA efficiency scores are relative estimates based on the observed sample and may be affected by unobserved contextual factors, such as regional economic development, policy implementation, demographic structure, and health care resource availability. Third, the study period overlapped with the COVID-19 pandemic, and the pandemic may have affected integrated medical and older adult care institutions differently across regions. Such regional differences in pandemic-related restrictions, service demand, staffing pressure, and resource allocation may have shifted the observed production frontier and introduced potential bias into the efficiency and productivity estimates. Therefore, the findings should be interpreted as relative short-term efficiency and productivity estimates within the observed sample, rather than as absolute measures of institutional performance. Future studies with larger samples and longer observation periods could further use bootstrapped DEA, second-stage regression, or meta-frontier approaches to examine the influence of contextual factors more explicitly.

## Conclusion

In this study, the total factor productivity (TFP) of 54 integrated medical and older adult care institutions in six provinces, namely, Shandong, Hebei, Hubei, Jilin, Inner Mongolia and Sichuan, was dynamically assessed by analyzing their data from 2020 to 2022 and applying the Malmquist production index under the DEA method. The results showed that total factor productivity fluctuated across the study period: it increased from 2020 to 2021 but declined from 2021 to 2022, reflecting the dynamic challenges faced by integrated medical and older adult care institutions. Regarding regional differences, integrated medical and older adult care institutions in the eastern region performed better than those in the central and western regions in terms of comprehensive efficiency and pure technical efficiency, which is closely related to the level of economic development, policy support, and the imbalance in the distribution of health care resources. In addition, some institutions, although showing high efficiency at the technical level, fail to reach the optimal level of comprehensive efficiency because of irrational scale allocation. This finding suggests that improving the efficiency of integrated medical and older adult care institutions not only relies on technological progress but also requires optimization in terms of resource allocation and scale management.

Improving the total factor productivity of integrated medical and older adult care institutions is a complex systematic task that requires policy support, resource allocation, technological innovation, and management optimization. In the future, the government should increase policy support and resource investment for integrated medical and older adult care institutions in the central and western regions to narrow regional efficiency gaps. Institutions should optimize internal management, rationally allocate medical, nursing, and rehabilitation resources, and strengthen cooperation with neighboring health care institutions to promote resource sharing and complementary advantages. Through coordinated efforts, integrated medical and older adult care institutions are expected to achieve higher productivity, provide better and more efficient services for older adults, and promote the sustainable development of integrated care.

## Data Availability

The data supporting the findings of this study are available from the corresponding author upon reasonable request.
